# Supplementary material for: A Global Dataset Mapping the AI Innovation from Academic Research to Industrial Patents
Source: Sci Data. 2025 Jul 18;12:1261. doi: 10.1038/s41597-025-05518-3 (PMC12274361; doi:10.1038/s41597-025-05518-3)
Supplement: Supplementary file 1 — Supplementary Information for: A Global Dataset Mapping the AI Innovation from Academic Research to Industrial Patents [file 41597_2025_5518_MOESM1_ESM.docx]

**Supplementary Information for:**

**A Global Dataset Mapping the AI Innovation from Academic Research to Industrial Patents**

**Supplementary Notes 1~3**

**Supplementary Figures 1~4**

**Supplementary Notes**

**Supplementary Note 1**

The academic papers in the DeepDiveAI.csv dataset were identified from the Intelligent Innovation Dataset (IIDS)[^1^](#IIDS2025)^,^[^2^](#Wu2025), comprising over 92.31 million academic records across diverse disciplines and publication formats. A hierarchical classification framework, tailored for scientific literature, was employed to identify 3,511,929 AI-related papers spanning 1961 to 2020. This process integrated expert knowledge with advanced deep learning models, including Long Short-Term Memory (LSTM), Qwen2.5 Plus (a large language model), and BERT-based classifiers. The methodology commenced with the construction of an expert-curated training dataset sourced from leading AI conferences (NeurIPS, ICLR, ICML) and non-AI disciplines, followed by LSTM-based coarse classification to generate a preliminary set of 9,479,846 potentially AI-related records. Subsequently, 10% of these records (947,985 entries) were randomly sampled and annotated using Qwen2.5 Plus to produce a refined labeled subset, which was used to train a BERT binary classifier for fine-grained classification. This approach was designed to address the unique challenges of scientific literature, such as interdisciplinary content, varied publication formats, and the evolving terminology of AI across research communities. While leveraging deep learning techniques similar to those commonly applied in patent classification, the academic paper classification was specifically adapted to the nuances of scholarly texts. The bibliographic sources include journal articles, conference proceedings, and other scholarly outputs from the IIDS database, ensuring comprehensive coverage of global AI research. For a detailed description of the methodology, validation results, and technical specifications, readers are referred to Zhou et al. (2024)[^3^](#Zhou2024).

**Supplementary Note 2**

AIfilter_Prompt = f"""

You are an Artificial Intelligence (AI) patent classifier. Your task is to determine whether each provided text, consisting of a patent title and abstract, is related to AI. If the text contains sensitive terms, skip analyzing those parts but still make an accurate classification based on the remaining content. Focus on identifying AI-related topics such as machine learning, deep learning, neural networks, natural language processing, computer vision, and other core AI technologies or applications. If the text lacks clear connections to AI, classify it as not related to AI.

Text: "{text}"

Please respond with only "True" or "False" without any additional explanation or labels.

"""

**Supplementary Note 3**

DeepInnovationAI enables comprehensive innovation analysis across multiple dimensions, including the following potential applications:

- **Global AI Competition Network Structure:** DeepInnovationAI enables global AI competition network analysis through Keywords, IPC that includes embedded geographic information, and semantic vector calculations from DeepPatentAI.csv. As illustrated in Fig. [S1](#Figure1), this network displays a clear "core-periphery" hierarchical structure, with the United States and China occupying dominant positions, while other countries remain at the periphery with comparatively limited technological development. The network's connection density and strength indicate both technological complementarity and competitive intensity between countries. These insights are valuable for shaping national innovation policies, guiding corporate strategies, and optimizing resource allocation in AI.
- **AI Technology Theme Analysis:** The DeepInnovationAI dataset enables comprehensive analysis of AI technology themes through structured terminology from the Keywords field in both DeepPatentAI.csv and DeepDiveAI.csv files. Fig. [S2](#Figure2) demonstrates how patent documents typically emphasize applications such as "methods," "neural networks," and "image processing," while academic papers focus more on fundamental theories like "deep learning." Such analyses facilitate the identification of technological frontiers and provision of empirical foundations for innovation strategy decisions.
- **AI Spatiotemporal Evolution Analysis:** The DeepInnovationAI dataset enables researchers to track how AI technologies evolve across time and geography by combining Year, Novelty, and IPC from DeepPatentAI.csv and DeepDiveAI.csv files. Fig. [S3](#Figure3) reveals exponential growth in overall AI activities, though with notable divergence in innovation patterns: patent innovation continues to grow steadily, while academic paper innovation shows a declining trend—potentially indicating the maturation and standardization of research paradigms. Geographic analysis highlights significant global imbalances in innovation activities, with the United States and China emerging as the predominant leaders in the field. This analytical framework provides valuable insights for tracking technological development trajectories, assessing the effectiveness of innovation policies, and forecasting future technological trends.


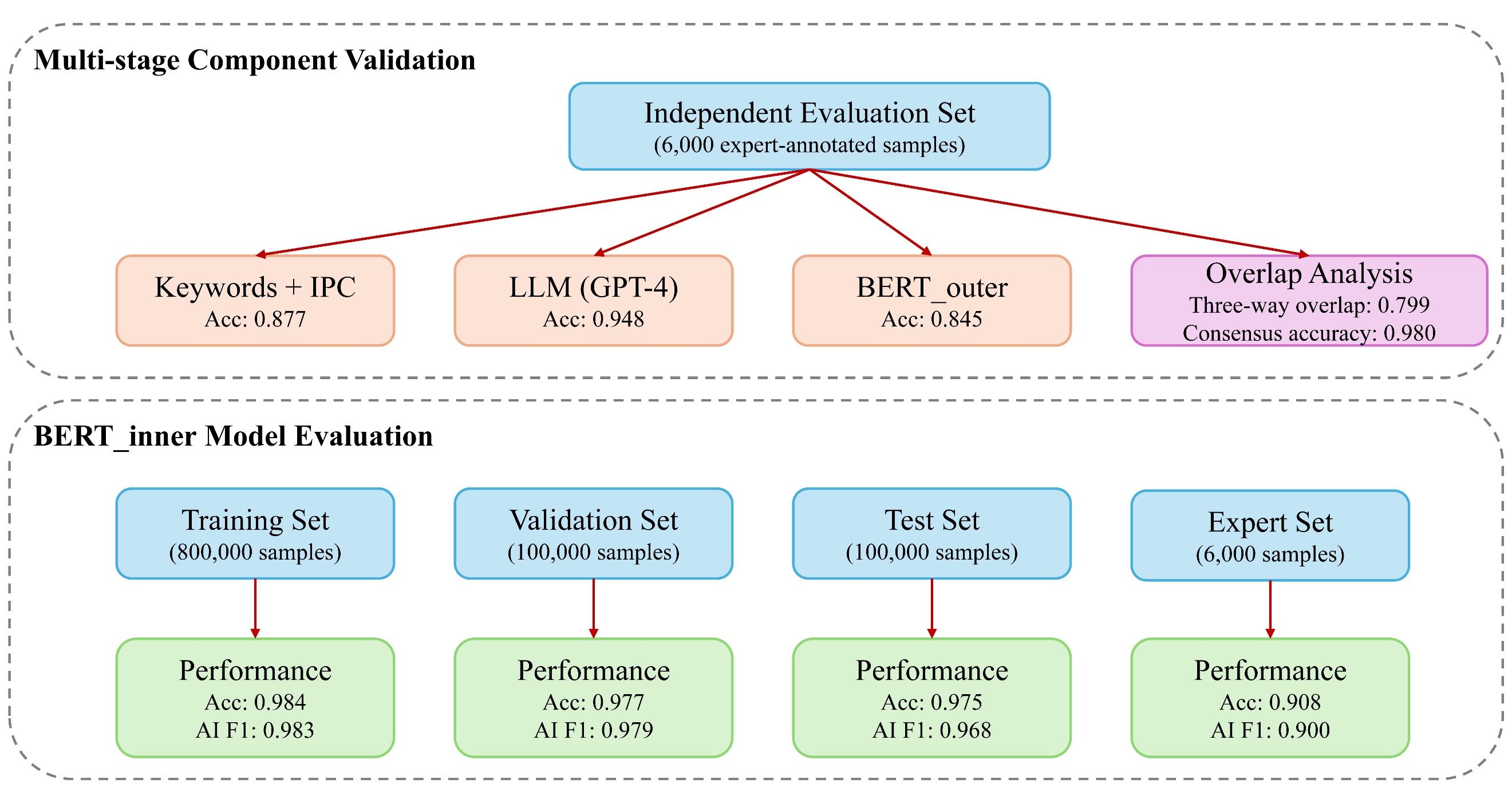


**Fig. S1** Technical validation workflow of the DeepInnovationAI dataset construction methodology showing multi-stage component validation and BERT_inner model evaluation across different datasets.


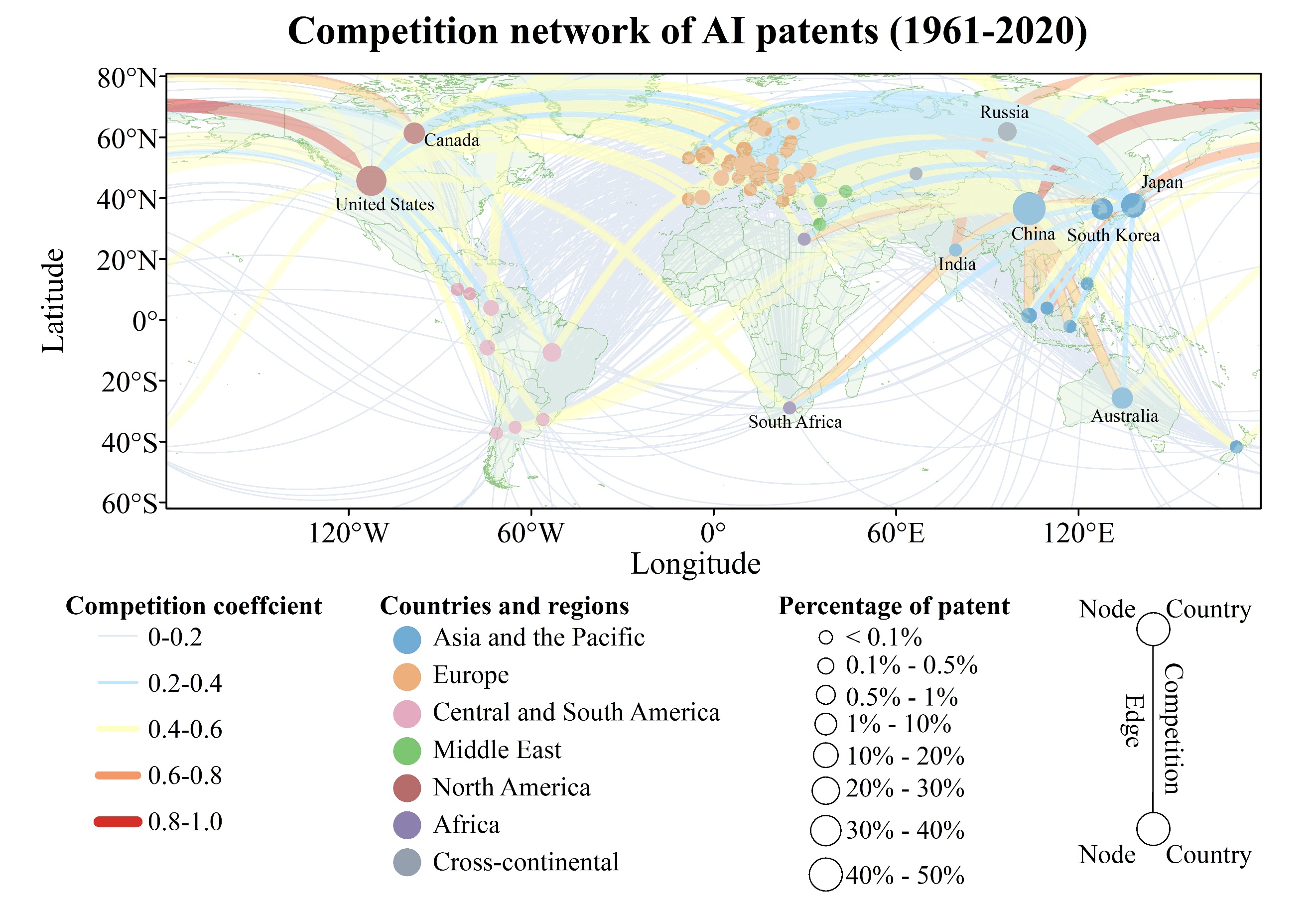


**Fig. S2** Network structure of global competition in artificial intelligence patents from 1961-2020. The competition coefficient is calculated based on the cosine similarity of patent topic vectors.


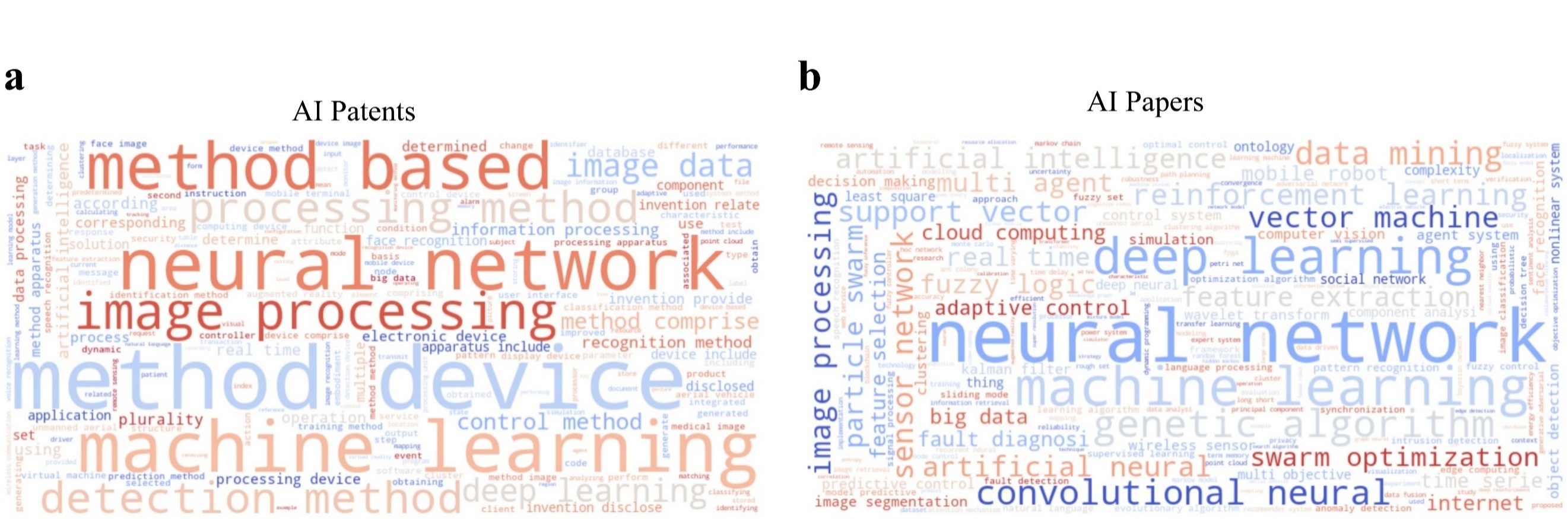


**Fig. S3** Word cloud diagram of the theme distribution of artificial intelligence patents and papers.


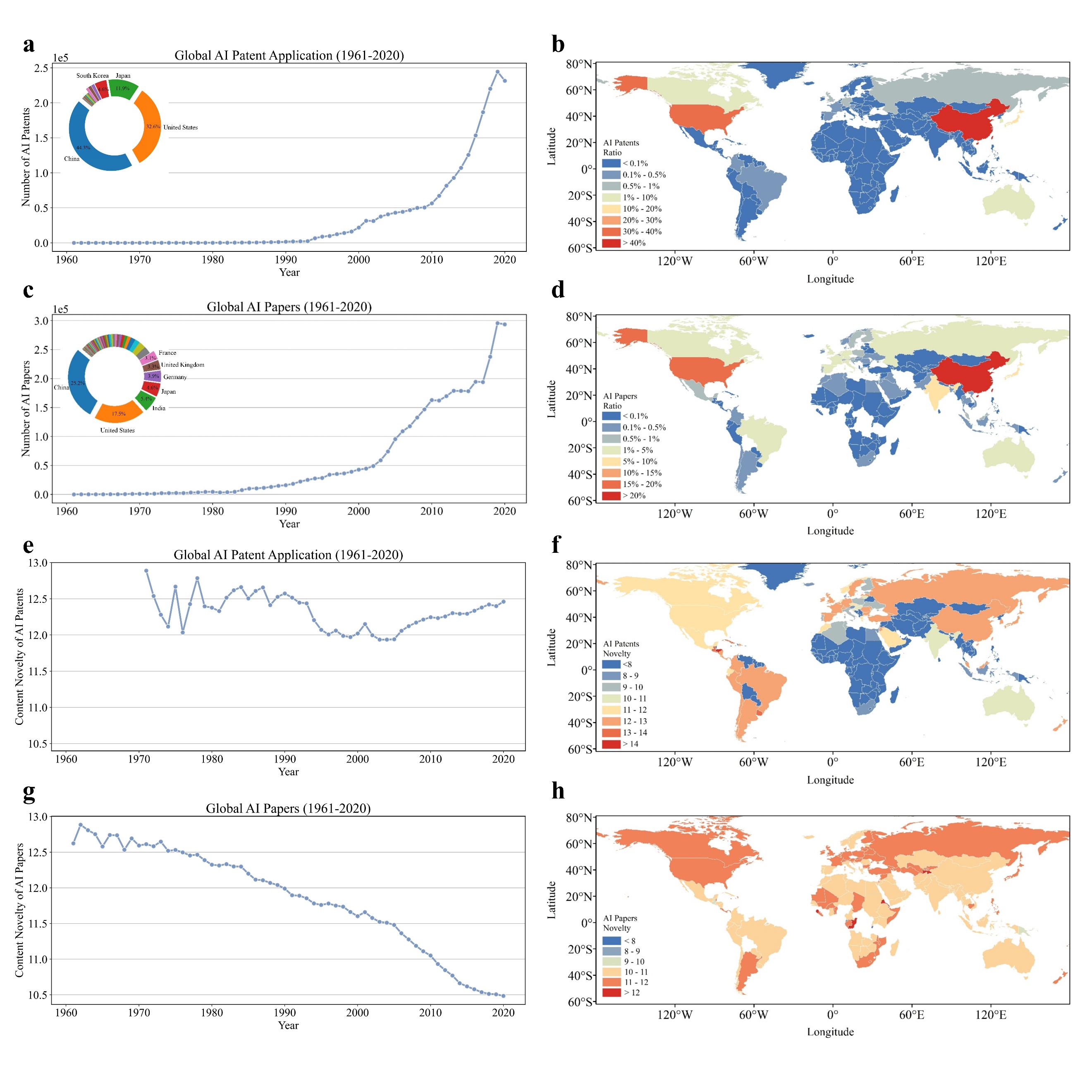


**Fig. S4** Spatiotemporal evolution characteristics of the **a-d** quantity and **e-h** content novelty of global DeepInnovationAI from 1961-2020.

**References**

1. IIDS. Intelligent Innovation Dataset. OpenDataLab <https://opendatalab.com/Gracie/IIDS> (2025).
2. Wu, X., Zou, H., Xing, Y. et al. Intelligent Innovation Dataset on Scientific Research Outcomes and Patents. J. Soc. Comput. 6, 63–73, <https://doi.org/10.23919/JSC.2024.0033> (2025).
3. Zhou, X., Liang, X., Zou, H., et al. DeepDiveAI: identifying AI related documents in large scale literature data. Preprint at <https://doi.org/10.48550/arXiv.2408.12871> (2024).
